# Supplementary material for: Regulation of Mitochondria-Associated Membranes (MAMs) by NO/sGC/PKG Participates in the Control of Hepatic Insulin Response
Source: Cells. 2019 Oct 25;8(11):1319. doi: 10.3390/cells8111319 (PMC6912364; doi:10.3390/cells8111319)
Supplement: Supplementary file 1 [file cells-08-01319-s001.pdf]

Supplementary data

Table S1: **Forward and reverse primers used in quantitative PCR on Huh7 cells.**

Common name	Official name	Forward	Reverse
GFP	*Gfp*	5'-ACG TAA ACG GCC ACA AGT TC-3'	5'-AAG TCG TGC TGC TTC ATG TG-3'
TBP	*Tbp*	5'-AGA CCA TTG CAC TTC GTG CC-3'	5'-CCT GTG CAC ACC ATT TTC CC-3'
COX-1	*Cox-1*	5'-ACT ATA CTA CTA CTA ACA GAC CG-3'	5'-GGT TCT TTT TTT CCG GAG TA -3'
PPIA	*Cyclophilin A*	5'-ACA CGC CAT AAT GGC ACT GG-3'	5'-CAG TCT TGG CAG TGC AGA T-3

Figure S1: eNOS is the main enzyme expressed and involved in NO synthesis in hepatocyte cells.

(A-B) Representative western-blot of the impact of modulating NO concentration using L-Name (1mM), arginine (1mM) and NONOate (1mM) on eNOS and iNOS expression in non-inflammatory conditions (A) *in vivo* in the liver of C57Bl6/JOlash male mice and (B) *in vitro* in Huh7 cells (control and CypD KO using CRISPR-cas9). Liver homogenate from a high fat-fed mouse was used as a positive control for iNOS.

(C) Representative image of eNOS and iNOS immunofluorescence on Huh7 cells and HUVEK cells (Human Umbilical Vein Endothelial Cells). Each red dots on the image corresponding to the eNOS or iNOS antibody labelling of each protein respectively. Cells were incubated overnight at 4°C with primary antibodies (1/500 dilution, Mouse, NOS3 sc-376751 or NOS2 sc-7271). After several washes with TBS-Tween 0.05%, cells were incubated for 1h at 37°C with the complementary secondary antibody (Alexa Fluor® 555, A21424). Cells were washed three times again with TBS-Tween 0.05% and mounted with "DuolinkII mounting medium" containing DAPI.

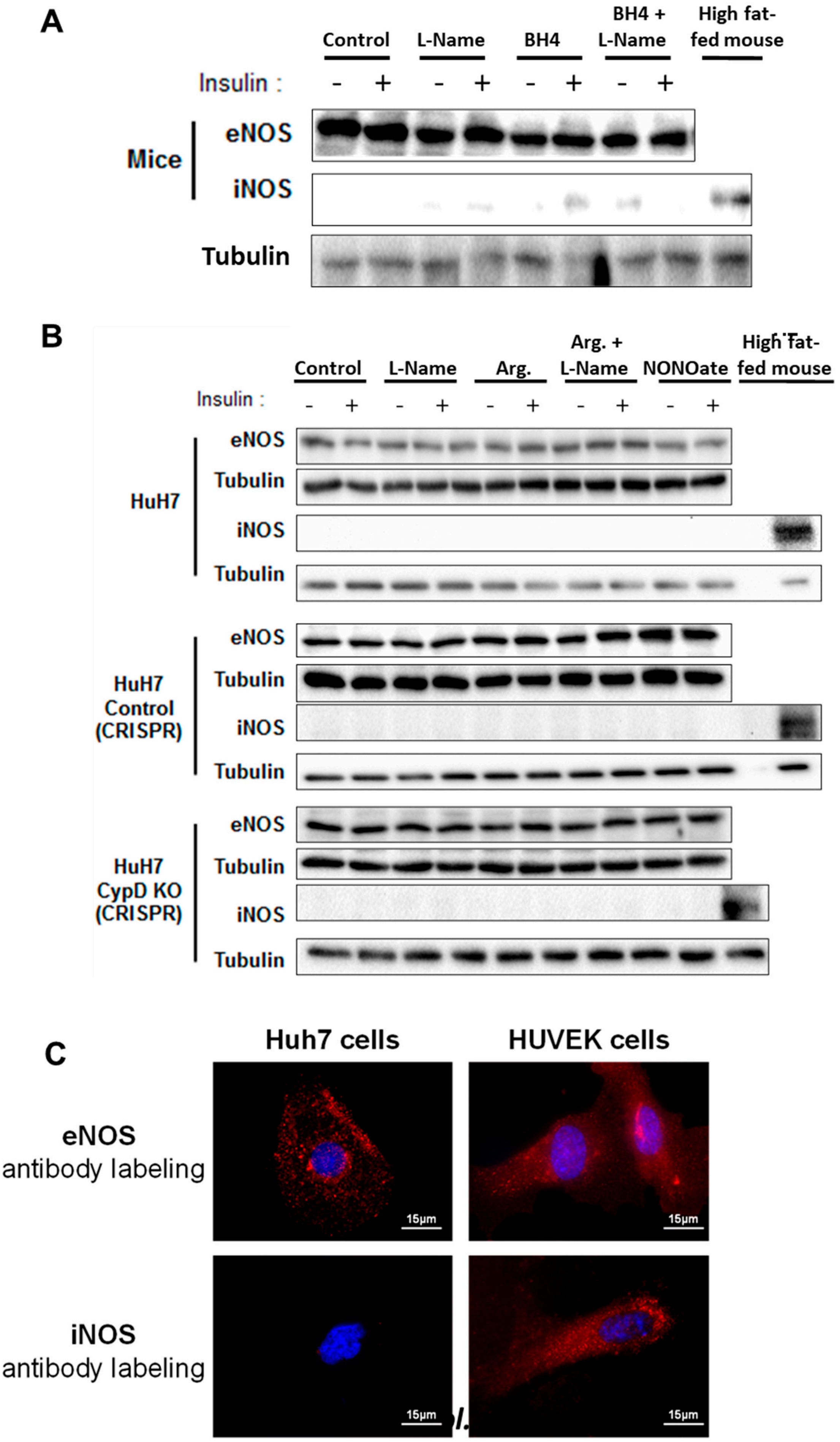
A
Control
L-Name
BH4
BH4 +
L-Name
High fat-
fed mouse
Insulin :
Mice
eNOS
iNOS
Tubulin
B
Control
L-Name
Arg.
Arg. +
L-Name
NONOate
High fat-
fed mouse
Insulin :
HuH7
HuH7
Control
(CRISPR)
HuH7
CypD KO
(CRISPR)
eNOS
Tubulin
iNOS
C
Huh7 cells
HUVEK cells
eNOS
antibody labeling
iNOS
antibody labeling
15µm

Figure S2:

(A) Purity of the fractions isolated from liver homogenates determined using immunoblotting for specific protein markers, i.e. aconitase for mitochondria, SIL-1 for ER, SERCA-2 for MAMs and VDAC1 for mitochondria and MAMs. C57Bl6/JOlash male mice received ip injection of BH4 (12.5mg/kg), L-Name (25mg/kg) and combination of the two, twice the day before and 2h before euthanasia. Representative western blots and commasie blue.

(B) Impact of modulating *in vivo* the NO production on protein content at the MAM fraction of key proteins involved in MAM formation, i.e. SERCA-2, mitofusin 2 (Mfn 2), glucose-regulated protein 75 (Grp75), cyclophilin D (CypD), VDAC1 and Optic atrophy type 1 (OPA1). Representative western blots, commasie blue and quantitative analyses (n = 5 mice per condition).

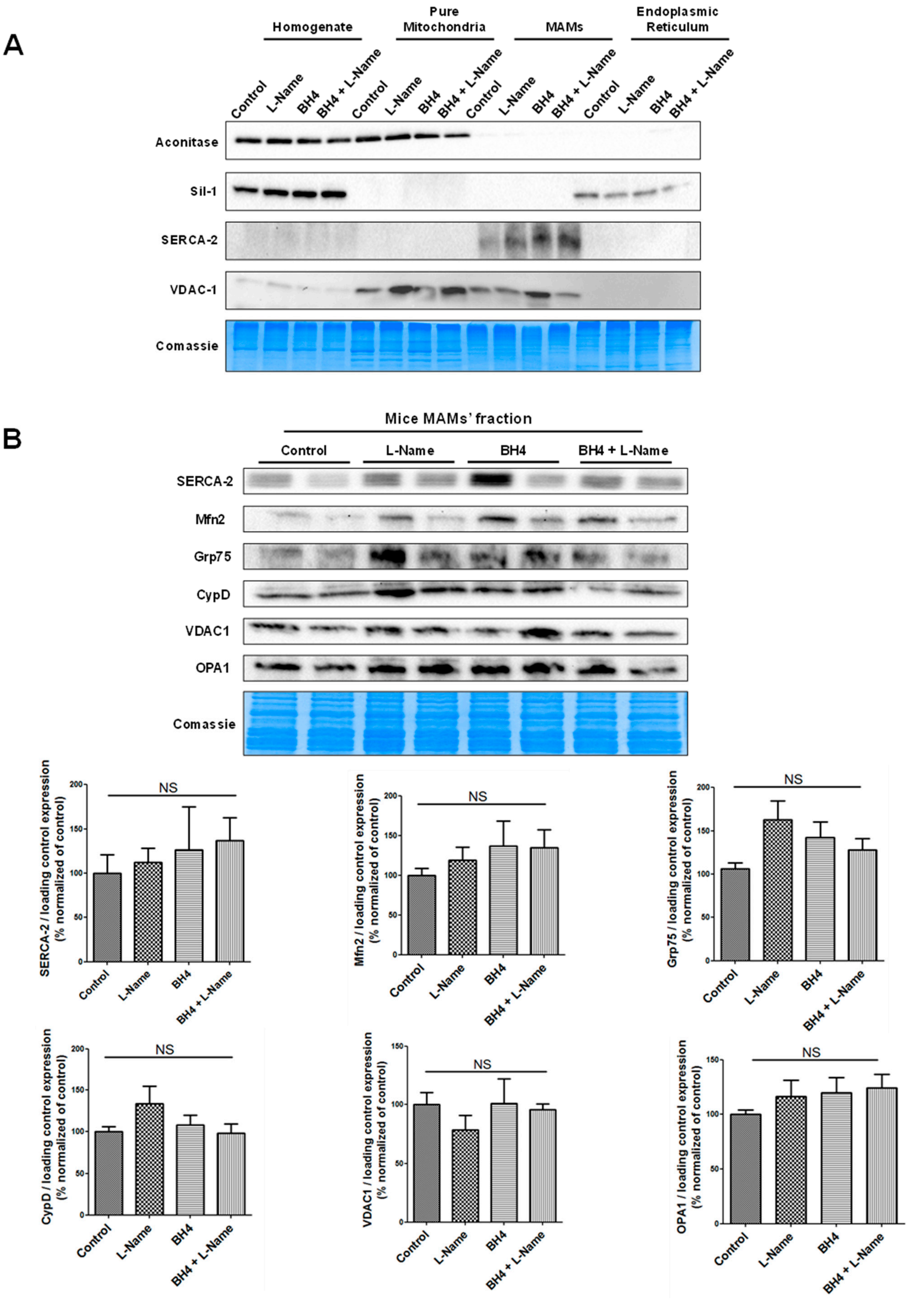
A
Homogenate
Pure Mitochondria
MAMs
Endoplasmic Reticulum
Control
L-Name
BH4
BH4 + L-Name
Aconitase
Sil-1
SERCA-2
VDAC-1
Comassie
B
Mice MAMs' fraction
Control
L-Name
BH4
BH4 + L-Name
SERCA-2
Mfn2
Grp75
CypD
VDAC1
OPA1
Comassie
SERCA-2 / loading control expression (% normalized of control)
Mfn2 / loading control expression (% normalized of control)
Grp75 / loading control expression (% normalized of control)
CypD / loading control expression (% normalized of control)
VDAC1 / loading control expression (% normalized of control)
OPA1 / loading control expression (% normalized of control)
NS

<u>Figure S3</u>:

(A) Impact of modulating *in vivo* the NO production on hepatic insulin signaling proteins located at MAMs. C57Bl6/JOlash male mice (n = 5 mice per condition) received ip injection of BH4 (12.5mg/kg), L-Name (25mg/kg) and combination of the two, twice the day before and 2h before euthanasia. Representative western blots and quantitative analyses of total and phosphorylated Akt and GSK3β according to treatments.

(B-C) Impact of arginine 1mM addition (B) or depletion after 24h treatment (C) during 5 to 60 minutes on ER-mitochondria interactions assessed through VDAC1/IP3R1 interactions using *in situ* PLA (n = 10 images minimum per experiment). In figure B, impact of 24h treatment with arginine 1mM is given as a reference. # and £, $p < 0.05$ vs. control (figure B) and Arginine 1 mM for 24 hours (figure C), respectively.

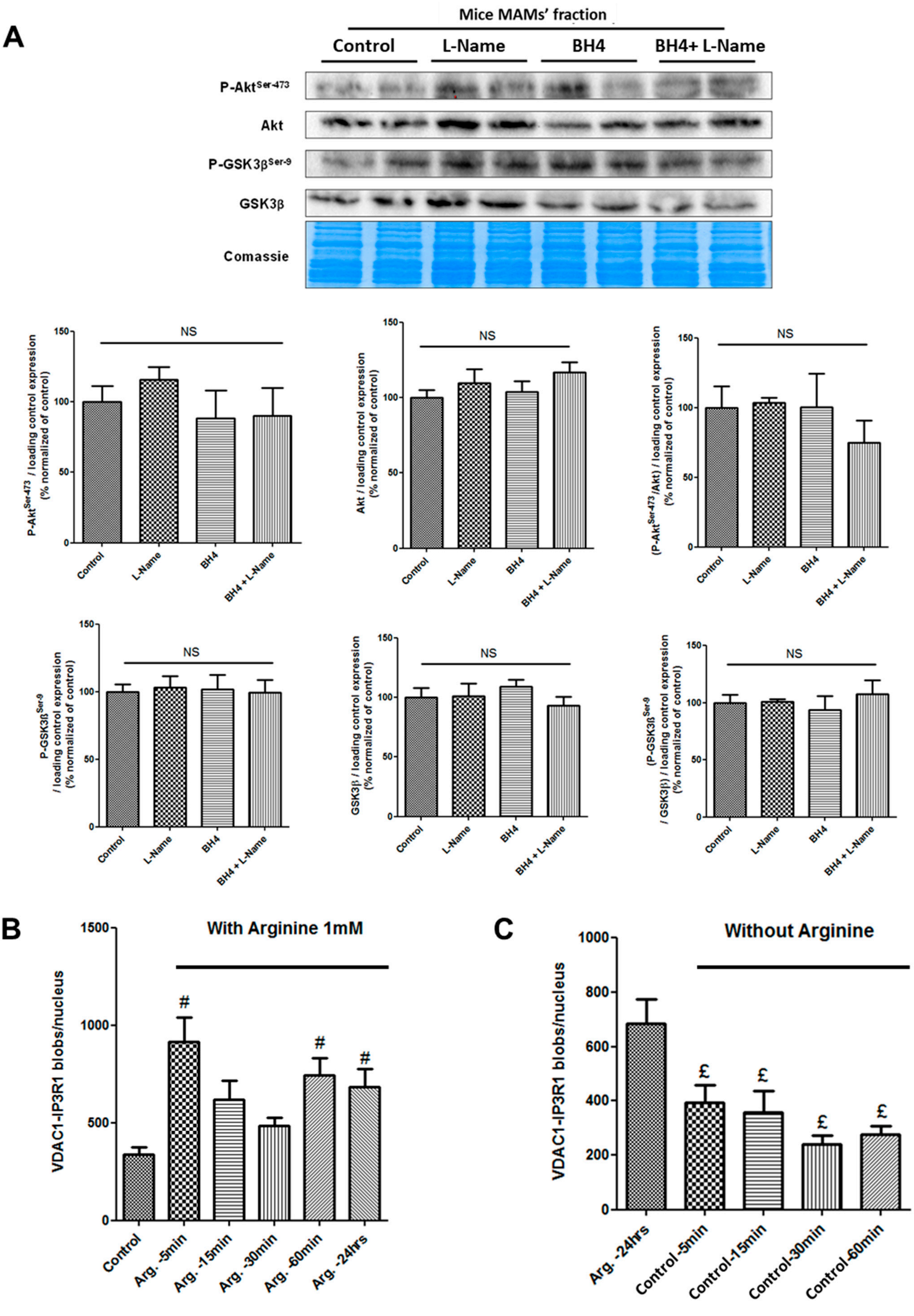
A
Mice MAMs' fraction
Control
L-Name
BH4
BH4+ L-Name
P-AktSer-473
Akt
P-GSK3βSer-9
GSK3β
Comassie
NS
P-AktSer-473 / loading control expression (% normalized of control)
Akt / loading control expression (% normalized of control)
(P-AktSer-473/Akt) / loading control expression (% normalized of control)
P-GSK3βSer-9 / loading control expression (% normalized of control)
GSK3β / loading control expression (% normalized of control)
(P-GSK3βSer-9 / GSK3β) / loading control expression (% normalized of control)
Control
L-Name
BH4
BH4 + L-Name
B
With Arginine 1mM
VDAC1-IP3R1 blobs/nucleus
Control
Arg. -5min
Arg. -15min
Arg. -30min
Arg. -60min
Arg. -24hrs
C
Without Arginine
VDAC1-IP3R1 blobs/nucleus
Arg. -24hrs
Control-5min
Control-15min
Control-30min
Control-60min

Figure S4:

(A) Representative Western blots of the impact of modulating NO production *in vitro* in Huh7 cells on protein content in VDAC1 and IP3R1.

(B) Cell viability in response to modulation of NO production assessed using Thoma cell counting chamber and automatic counting using the LUNA^{TM} Automated Cell Counter (Logos Biosystems). Representative images are presented on the next page.

(C) Representative Western blots of the impact of modulating NO production *in vitro* in Huh7 cells on protein content of caspase 3, a marker of apoptosis.

(D) Representative Western blots of the impact of modulating NO production *in vitro* in Huh7 cells on protein content in markers of the endoplasmic reticulum stress.

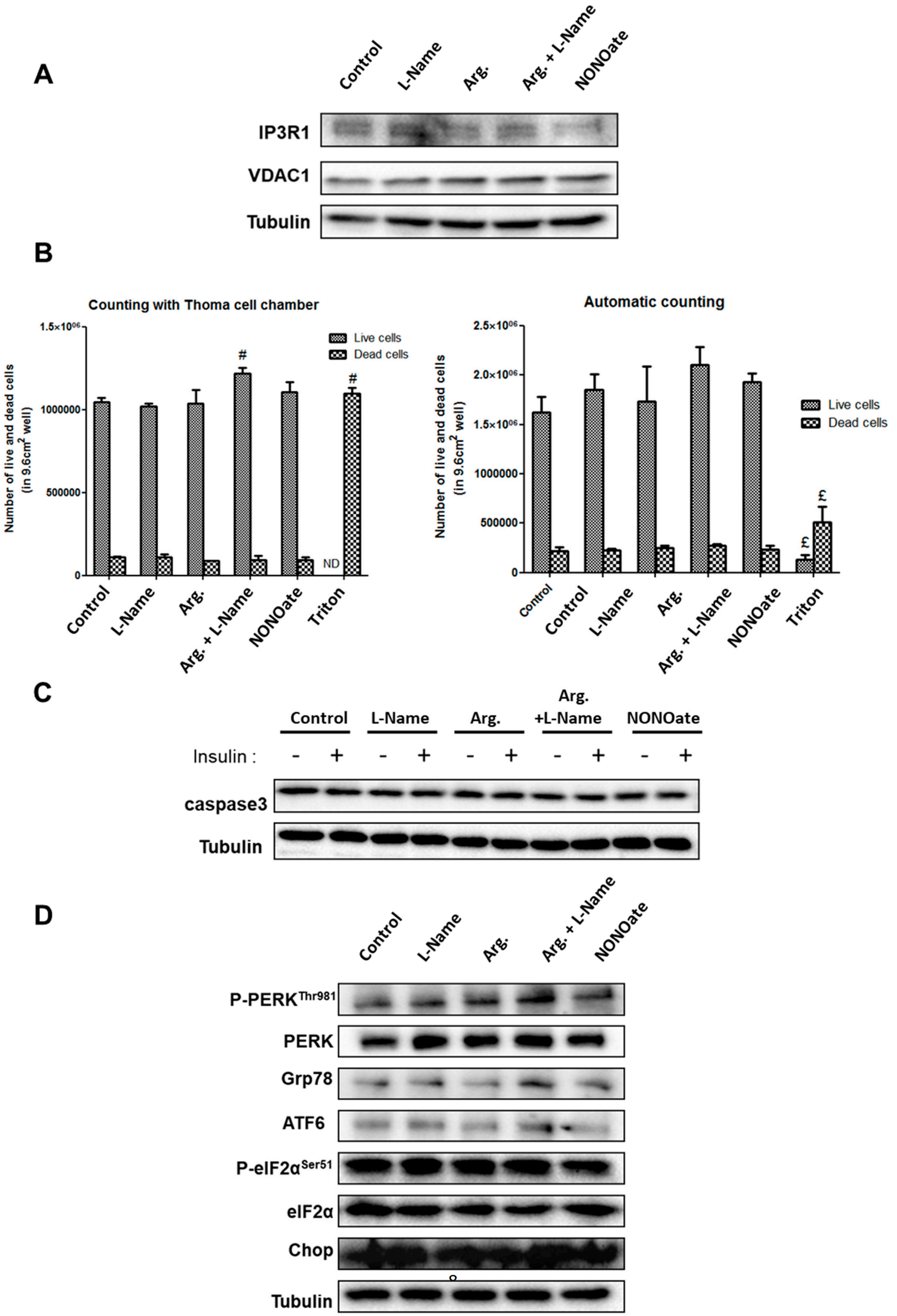
A
Control
L-Name
Arg.
Arg. + L-Name
NONOate
IP3R1
VDAC1
Tubulin
B
Counting with Thoma cell chamber
Number of live and dead cells (in 9.6cm2 well)
Live cells
Dead cells
#
ND
Triton
Automatic counting
C
Arg. +L-Name
Insulin :
caspase3
D
P-PERKThr981
PERK
Grp78
ATF6
P-eIF2αSer51
eIF2α
Chop

Huh7 before trypsine (in 9,6cm2 well)

Huh7 after trypsine and stain with blue trypan in Thoma chamber

Huh7 after trypsine and stain with blue trypan in Automatic Logos Biosystems

Control

L-Name

Arg.

Arg. + L-Name

NONOate

Control + Tryton

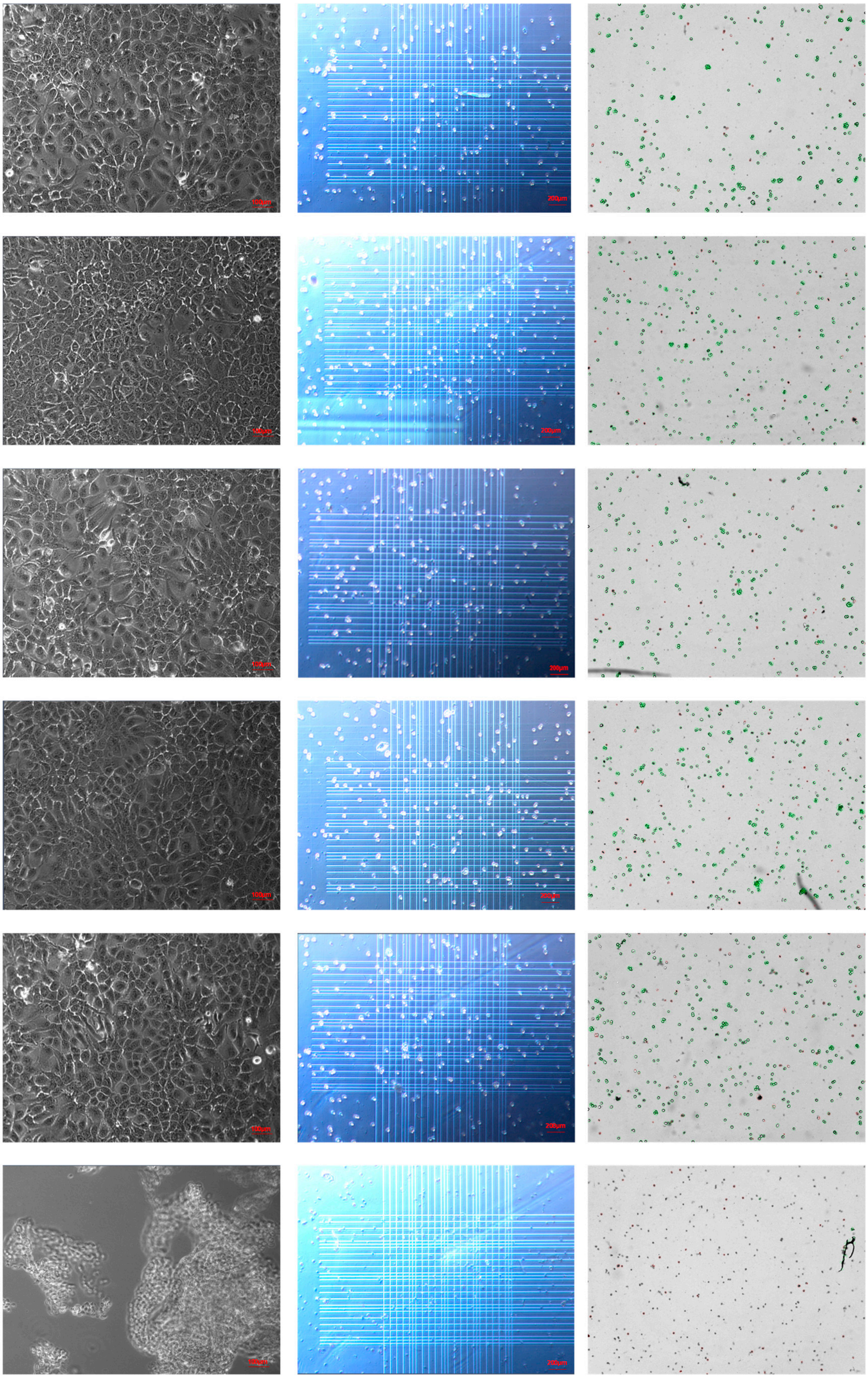

Figure S5:

(A) Impact of modulating *in vitro* NO production on whole cell oxygen consumption assessed using oxygraphy in primary rat hepatocytes (n = minimum 6 independent experiments). Oxygen consumption was monitored in intact primary rat hepatocytes in DMEM medium with glucose and treatments. Basal respiration was assessed at 37°C with treatments. Addition of 10µg/mL of oligomycin provided uncoupled (non-phosphorylating) oxygen consumption.

(B) Representative scheme of Wortmannin action on the molecular Akt signalization.

(C-E) Impact of L-Name (1mM), arginine (1mM), and combination of the two on NO concentration assessed using Daf-FM (15µM in PBS, 30 min at 37°C) in fresh Huh7 cells (C) treated with Wortmannin 1µM for one hour, (D) CRISPR control and KO CypD CRISPR (clone F1 used in figure 7 of this article), and (E) adCherry (as adGFP would interefer with Daf-FM signal) and adFate-1 transfected cells.

£ and #, $p < 0.05$ vs. control; ***, $p < 0.05$ vs. respective uncoupled state.

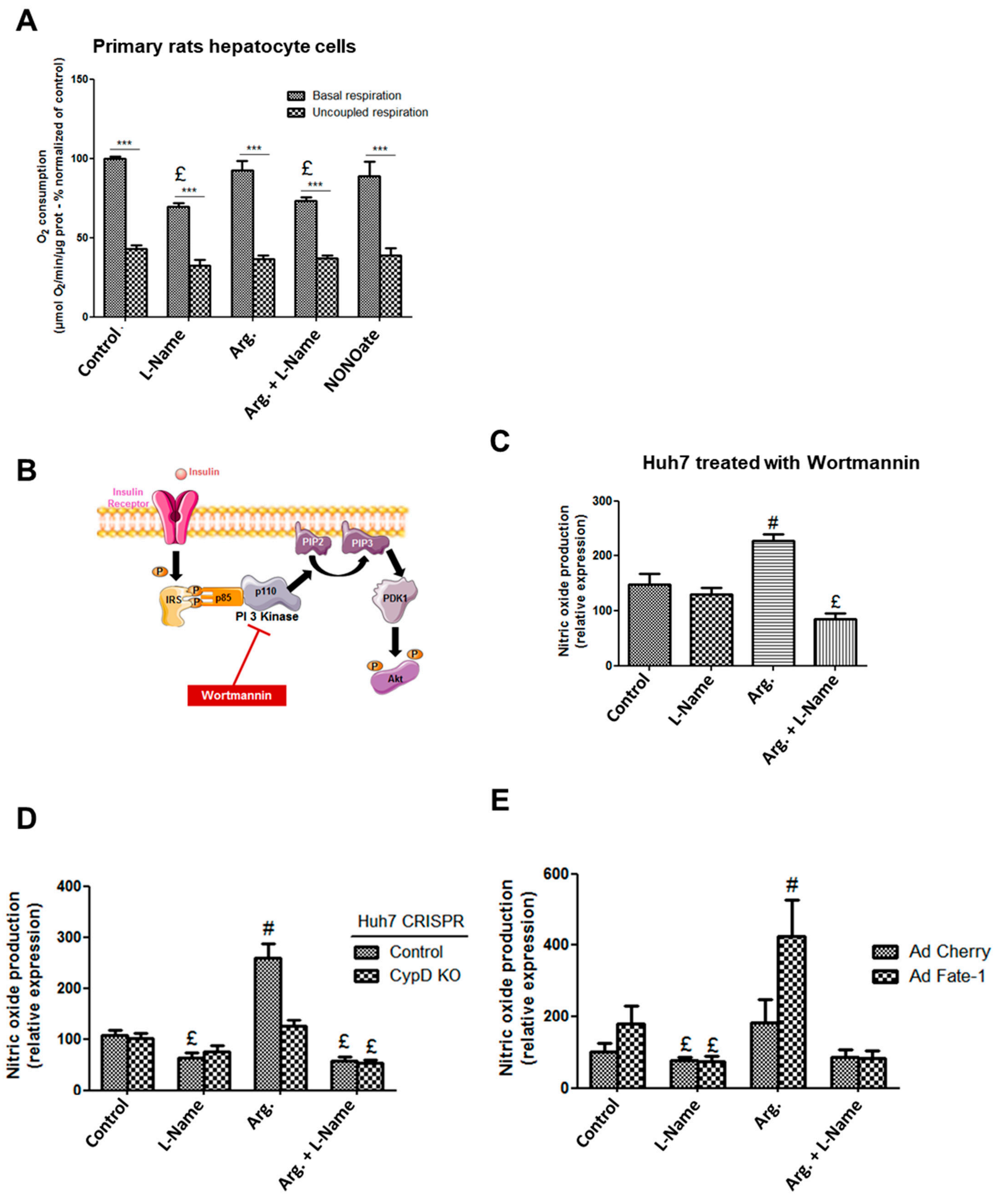
A
Primary rats hepatocyte cells
O2 consumption (µmol O2/min/µg prot - % normalized of control)
Basal respiration
Uncoupled respiration
Control
L-Name
Arg.
Arg. + L-Name
NONOate
B
Insulin
Insulin Receptor
IRS
p85
p110
PI 3 Kinase
PIP2
PIP3
PDK1
Akt
Wortmannin
C
Huh7 treated with Wortmannin
Nitric oxide production (relative expression)
Control
L-Name
Arg.
Arg. + L-Name
D
Huh7 CRISPR
Control
CypD KO
Nitric oxide production (relative expression)
E
Ad Cherry
Ad Fate-1
Nitric oxide production (relative expression)

<u>Figure S6</u>: CypD downregulation blunts NO impact on VDAC1/IP3R1 interaction.

(A) Representative Western blots (at top) and quantitative analysis (below) of CypD level protein expression on Huh7 cells Control and CypD KO in three different clone using the CRISPR-cas9 technical (the clone F1 corresponding of the Huh7 CypD KO clone use for the figure 7 of this article).

(B) Impact of modulating NO concentration using L-Name (1mM), arginine (1mM) and Nonoate (1mM) in Huh7 cells control or KO CypD CRISPR-cas9 (clone E2 is an another clone of KO cells different from the one used in figure 7 of this article) on ER-mitochondria interactions assessed through VDAC1/IP3R1 interactions using *in situ* proximity ligation assay (PLA).

(C) Impact of NO substrate (arginine, 1mM) or donor (Nonoate, 1mM) on Huh7 cells transfected with siRNA control (in black) or CypD (in grey) on ER-mitochondria interactions assessed through VDAC1/IP3R1 interactions using *in situ* PLA.

For *in situ* PLA, representative image (at top) and quantitative analysis of VDAC1/IP3R1 interactions (below) (n = 10 images minimum per experiment, 3 independent series per treatment, scale bar 15µm, x40 or x100). For siRNA transfection, cells were transfected with CypD siRNA (25nmol/L) using the transfection reagent HiPerFect following the technical recommendations (Qiagen). Medium was changed 24h after transfection and experimentations were performed 48h later. £ and #, $p < 0.05$ vs. control; ***, $p < 0.05$ vs. respective treated control.

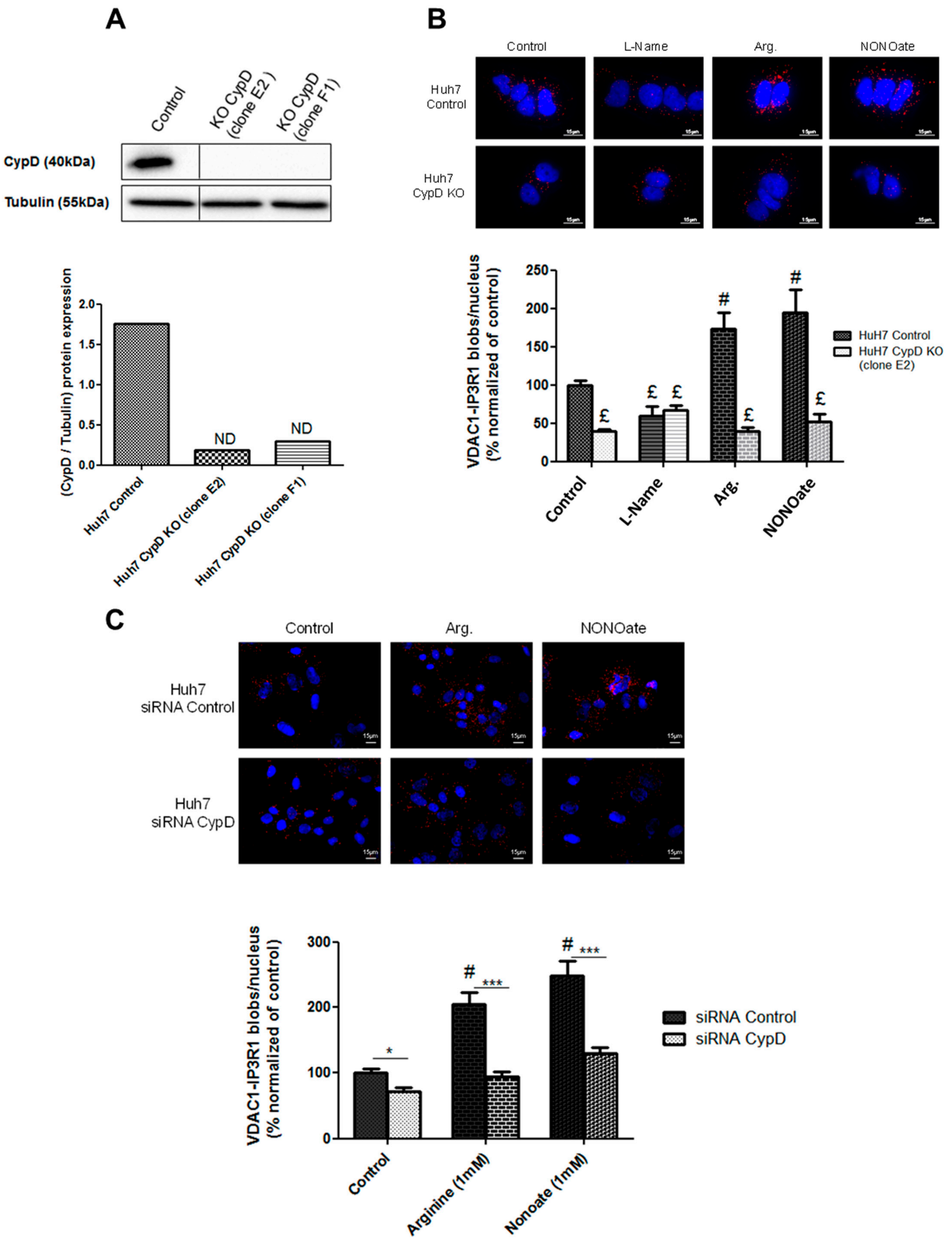
A
Control
KO CypD (clone E2)
KO CypD (clone F1)
CypD (40kDa)
Tubulin (55kDa)
(CypD / Tubulin) protein expression
2.0
1.5
1.0
0.5
0.0
ND
ND
Huh7 Control
Huh7 CypD KO (clone E2)
Huh7 CypD KO (clone F1)
B
Control
L-Name
Arg.
NONOate
Huh7 Control
Huh7 CypD KO
VDAC1-IP3R1 blobs/nucleus (% normalized of control)
250
200
150
100
50
0
HuH7 Control
HuH7 CypD KO (clone E2)
Control
L-Name
Arg.
NONOate
C
Control
Arg.
NONOate
Huh7 siRNA Control
Huh7 siRNA CypD
VDAC1-IP3R1 blobs/nucleus (% normalized of control)
300
200
100
0
siRNA Control
siRNA CypD
Control
Arginine (1mM)
Nonoate (1mM)

Figure S7: Enlarged representative images presented in Figure 1 to 7. Original figure number is mentioned in title.

A- Fig. 3C

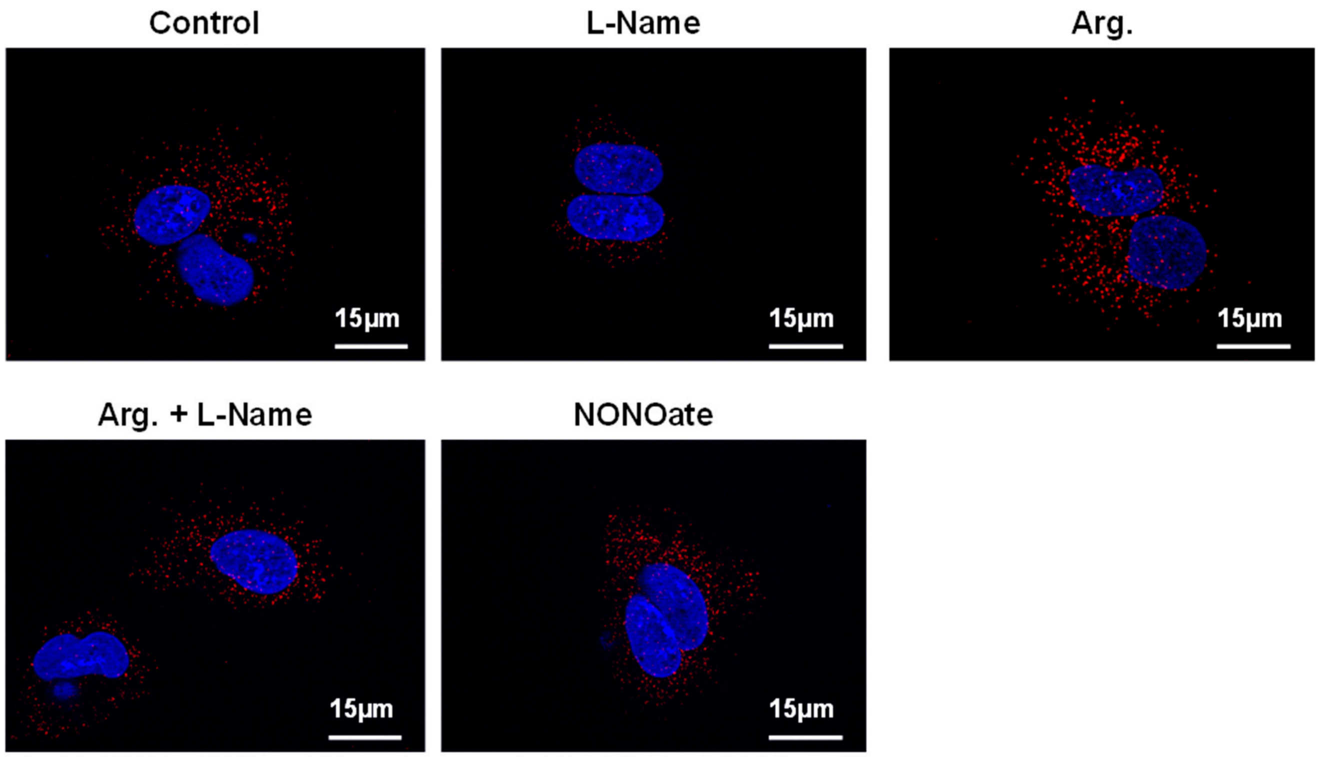

B- Fig. 3D

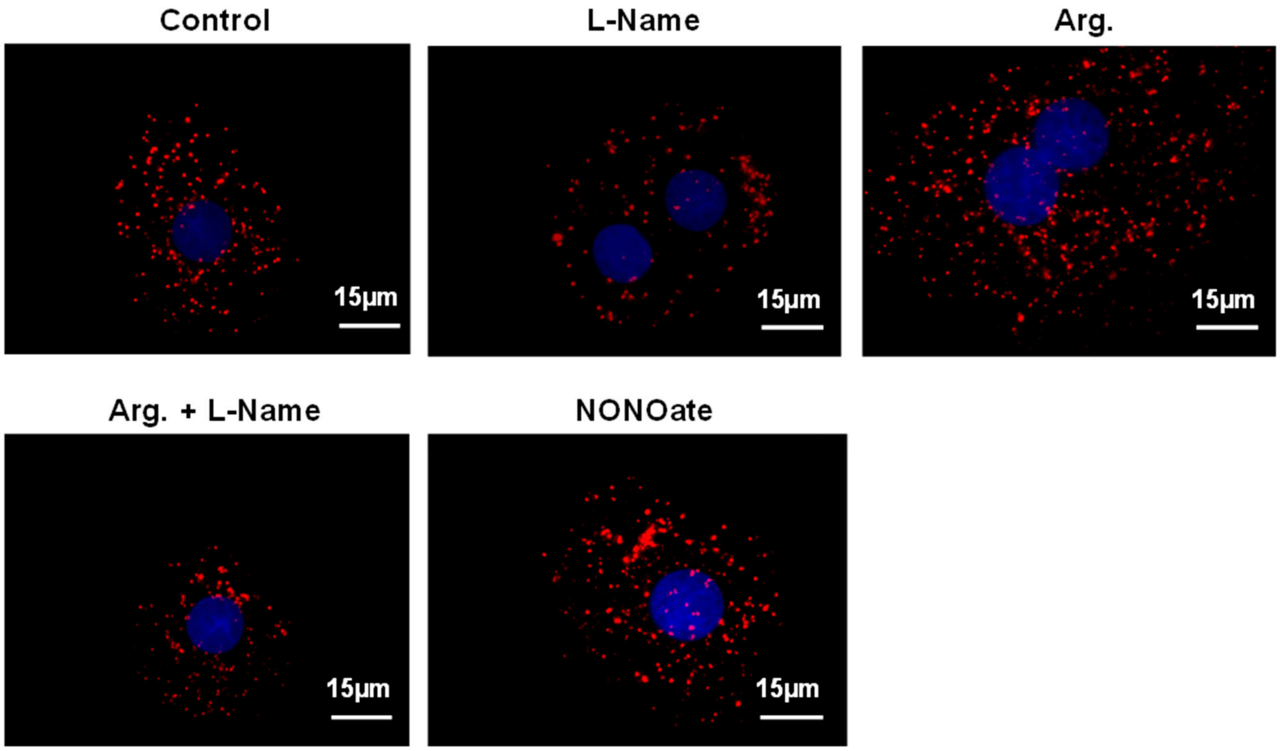

C- Fig. 3E

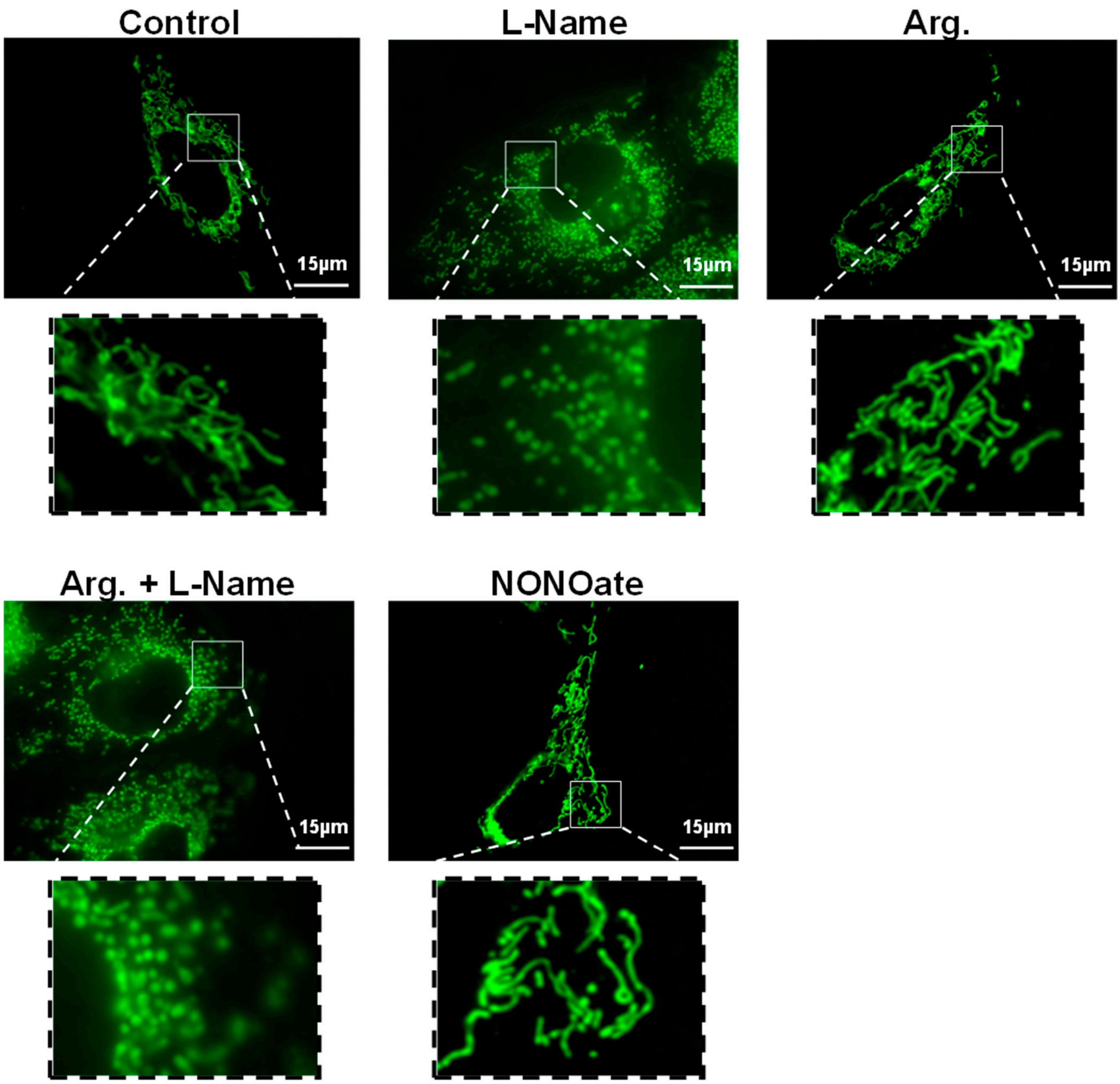
Control
L-Name
Arg.
15µm
15µm
15µm
Arg. + L-Name
NONOate
15µm
15µm

D- Fig. 4C

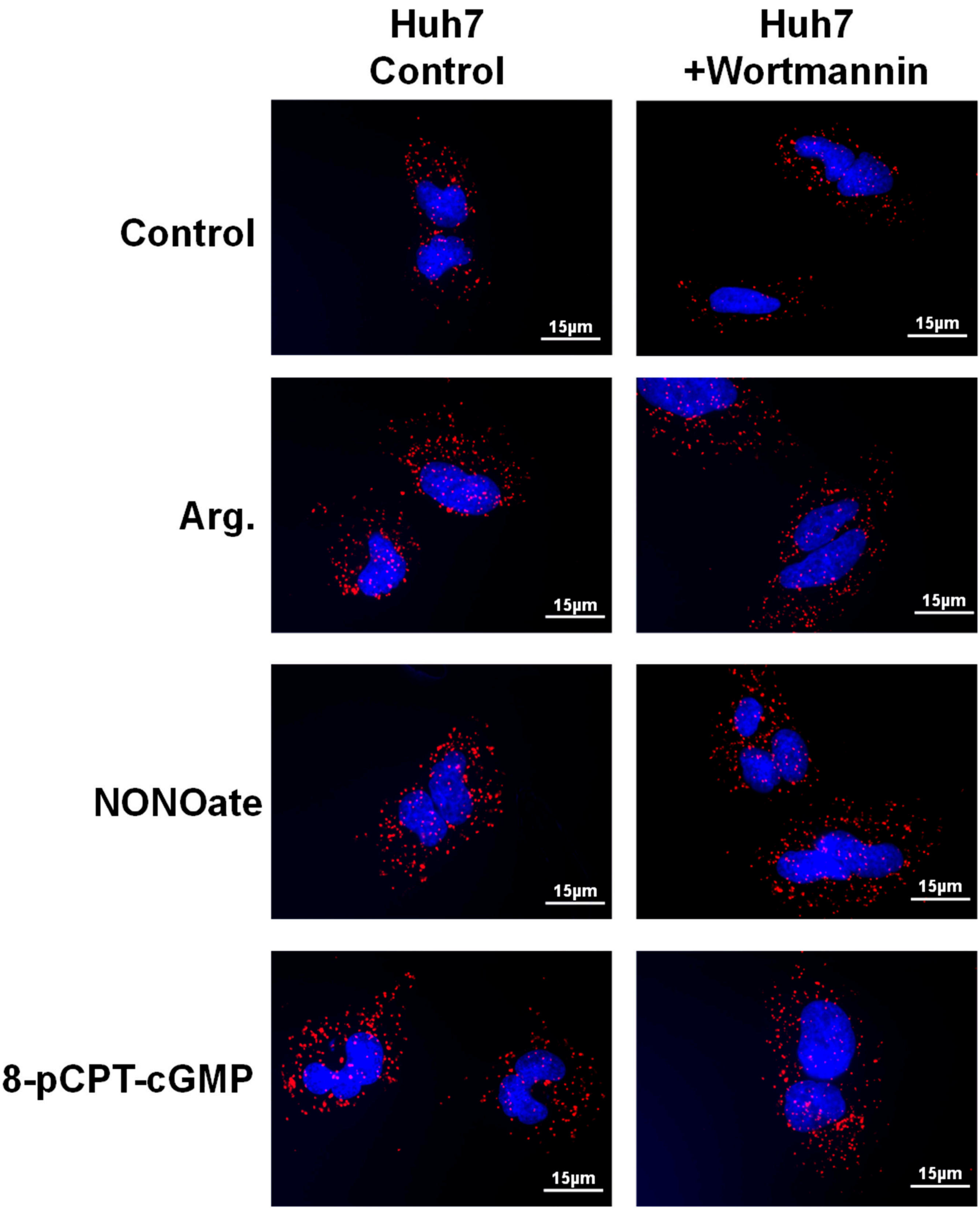
Huh7
Control
Huh7
+Wortmannin
Control
Arg.
NONOate
8-pCPT-cGMP
15µm
15µm
15µm
15µm
15µm
15µm
15µm
15µm

E- Fig. 5B

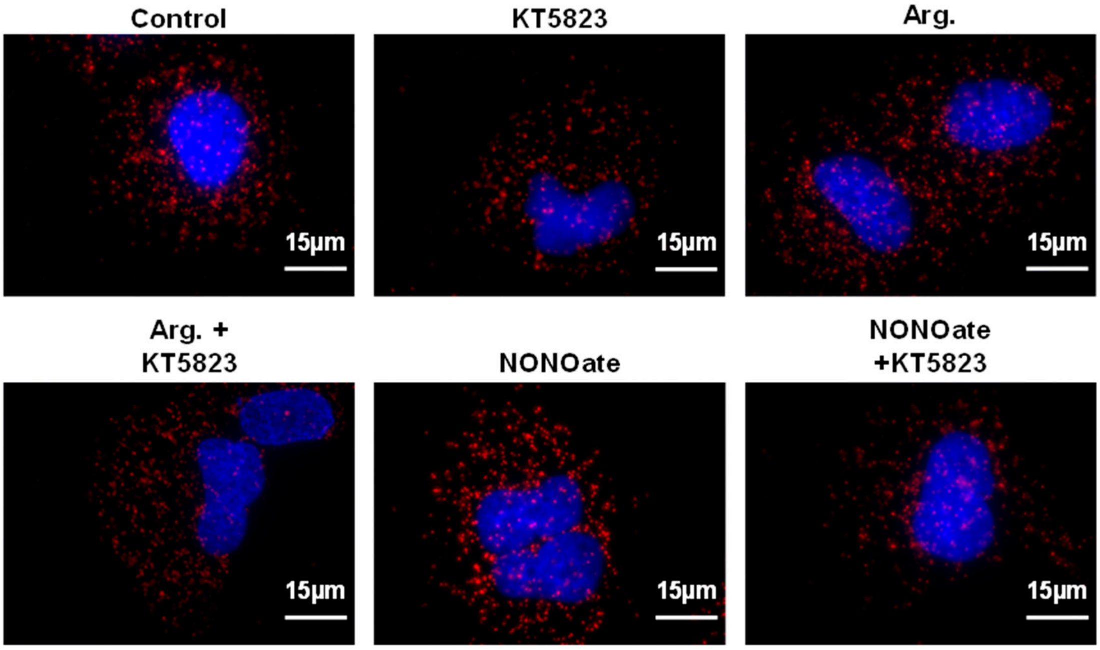

F- Fig. 5C

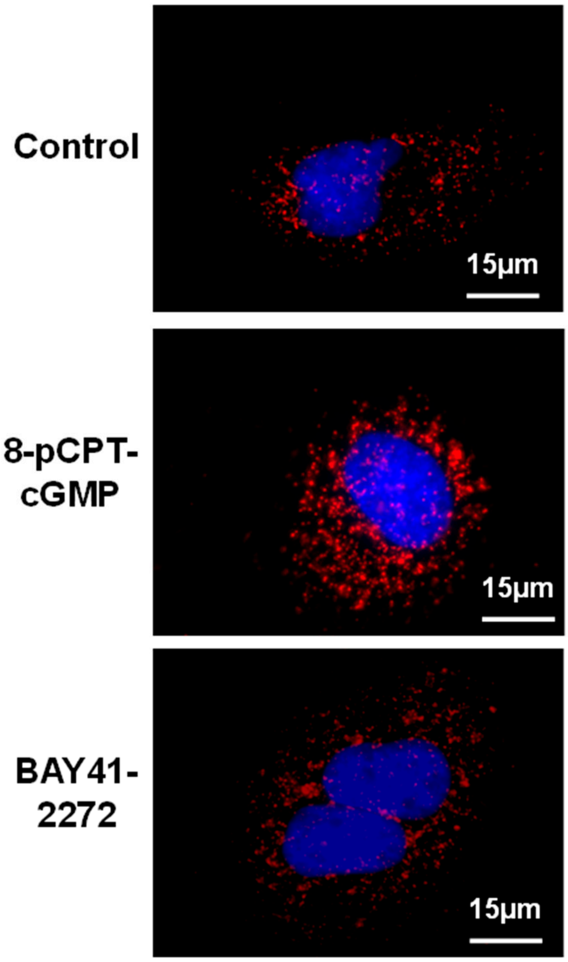

G- Fig. 5D

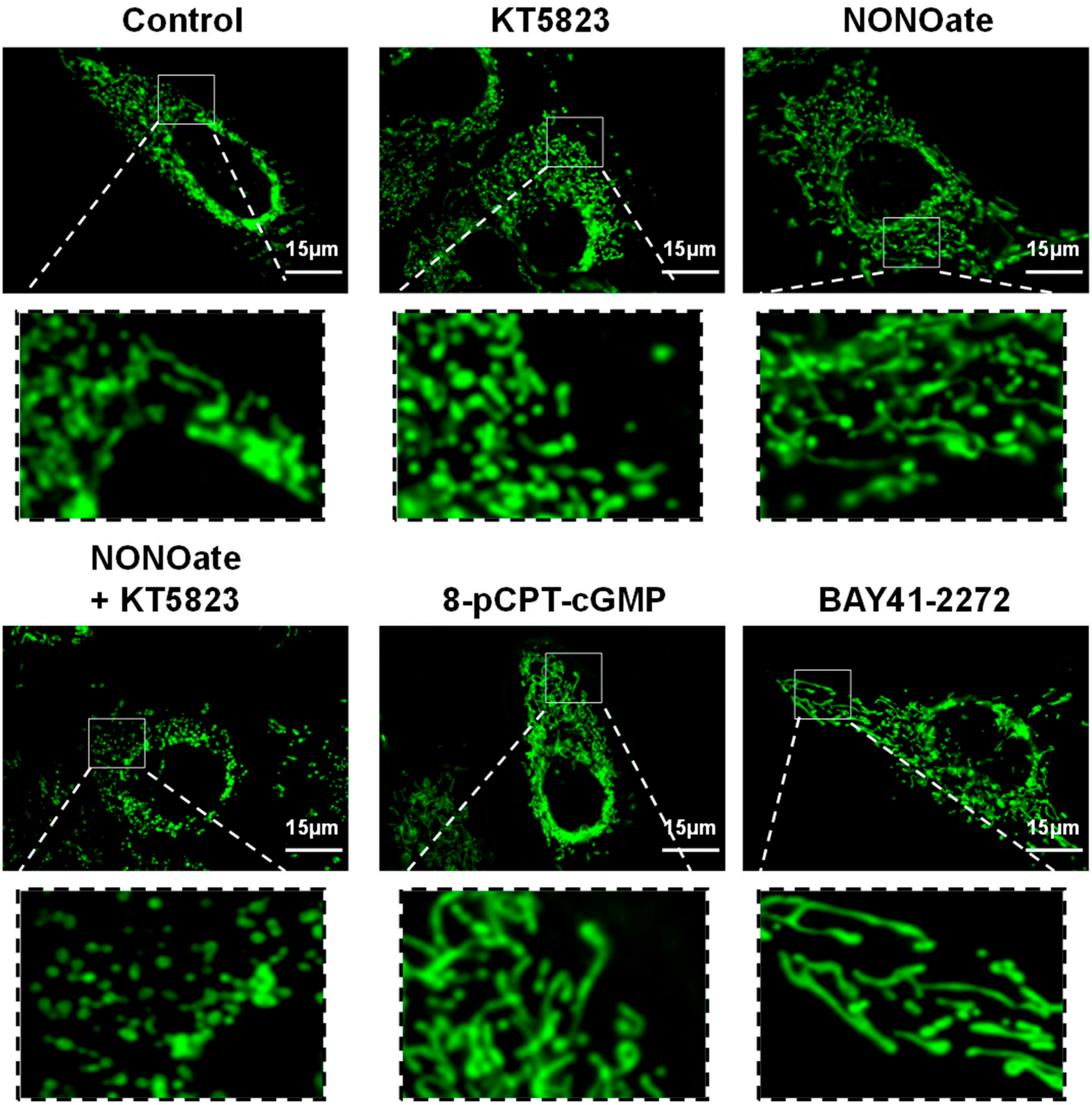

Control
KT5823
NONOate
15µm
15µm
15µm
NONOate + KT5823
8-pCPT-cGMP
BAY41-2272
15µm
15µm
15µm

H- Fig. 7A

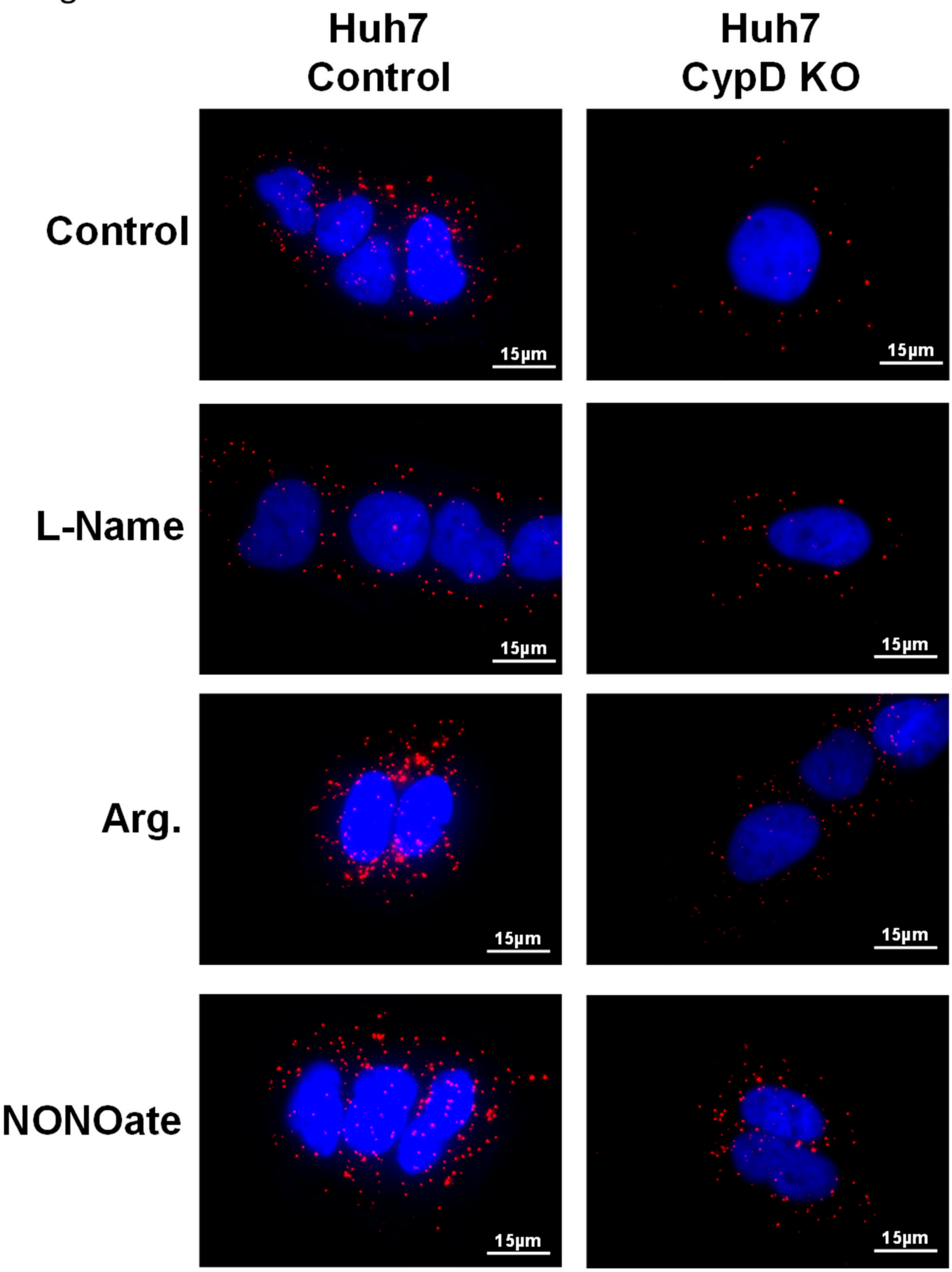
Huh7
Control
Huh7
CypD KO
Control
L-Name
Arg.
NONOate
15µm
15µm
15µm
15µm
15µm
15µm
15µm
15µm

I- Fig. 7B

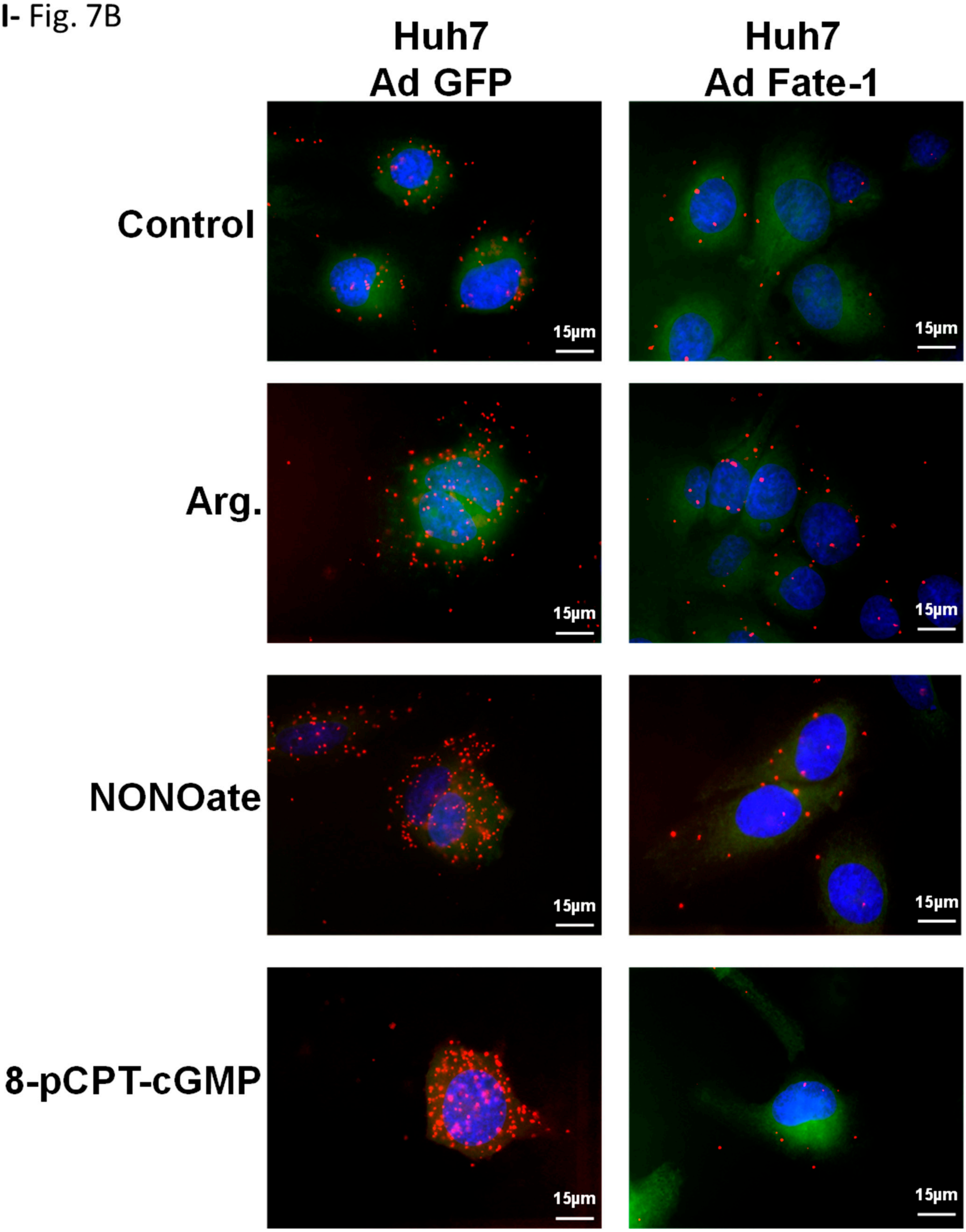
Huh7
Ad GFP
Huh7
Ad Fate-1
Control
Arg.
NONOate
8-pCPT-cGMP
15μm
